# Differential production of prostaglandins and prostacyclins by liver and head kidney cells from Atlantic salmon challenged with arachidonic and eicosapentaenoic acids

**DOI:** 10.1016/j.fsirep.2021.100015

**Published:** 2021-07-03

**Authors:** Pedro Araujo, Marit Espe, Eva Lucena, Yang Yang, Elisabeth Holen

**Affiliations:** a Institute of Marine Research (HI), PO Box 1870 Nordnes, N-5817 Bergen, Norway; b Department of Chemistry, Organic Analysis and Catalysis Laboratory, Simon Bolivar University, Caracas 1080A, Venezuela; c Key Laboratory of Food and Biological Engineering of Zhejiang Province, Hangzhou Wahaha Group Co.Ltd, Hangzhou 310018, China

**Keywords:** prostaglandins, prostacyclins, arachidonic acid, eicosapentaenoic acid, signalling pathways, Atlantic salmon, cell culture, liver cells, head kidney cells

## Abstract

Polyunsaturated fatty acids such as arachidonic and eicosapentaenoic acids are the precursors of eicosanoid metabolites (e.g prostaglandins and prostacyclins) which regulate inflammatory and immune response processes in fish organs. The present research studies the differential production of PGI_2_, PGI_3_, PGE_2_ and PGE_3_ by primary liver and head kidney cells isolated from salmon and challenged with single or combined ARA and/or EPA. There was a significant increase in the production of PGE_2_ and PGI_3_ in both types of cells after exposure to single and combined fatty acids. Increased production of PGE_3_ was only detected in liver cells after exposure to ARA+EPA. The levels of PGI_2_ in liver cells were significantly increased after exposure to all the tested fatty acid systems, while the production levels in head kidney cells were only significant after exposure to ARA or ARA+EPA, but not to EPA, where the production was non-significantly decreased compared to the control cells. In general, liver cells synthetized higher prostaglandin levels than prostacyclins, and the opposite was observed in head kidney cells, which synthetized highly remarkable amounts of prostacyclin compared to liver cells. The overall production for both types of cells and various fatty acid systems were characterized by a high proportion of the omega-3 fatty acid metabolites (PGE_3_+PGI_3_) compared to the omega-6 counterpart (PGE_2_+PGI_2_). Some potential production mechanisms are proposed and comprehensively discussed. The results of the present research are the first to deliver the differential production of prostacyclins and prostaglandins by liver and head kidney cells from salmon, thereby paving the way for understanding the significance of these prostanoids in fish physiology and disease.

## Highlights

• Head kidney cells synthetized considerable higher levels of prostacyclins (PGI) than prostaglandins (PGE), while the liver cells exhibited the opposite behaviour.
• Head kidney cells synthetized highly remarkable amounts of PGI_2_ and PGI_3_ compared to liver cells.
• The levels omega-3 fatty acid metabolites (PGE_3_+PGI_3_) were consistently higher than omega-6 fatty acid metabolites (PGE_2_+PGI_2_) in both types of cells and tested preparations.
• Potential production mechanisms are proposed and discussed.

## 1 Introduction

Studies on prostaglandins and prostacylcins in fish and mammals started over 45 years ago when Nomura et al. [1] and Bunting et al. [2] isolated PGE_2_ and PGI_2_ from fish (flounder and tuna) testis and rabbit arteries, respectively. In general, prostanoid research has been more focused on mammalian than fish models, hence the regulation of prostanoid metabolites and involvement in different physiological processes remains poorly characterized in fish.

Arachidonic acid (ARA; 20:4n-6) and eicosapentaenoic acid (EPA; 20:5n-3) are the precursors of pro-inflammatory and anti-inflammatory prostanoids, respectively. The synthesis of pro-inflammatory prostanoids, such as prostaglandin E2 (PGE_2_) and prostacyclin I2 (PGI_2_) consist of releasing ARA from the cellular membrane, followed by the catalysis of the cyclooxygenase enzyme to render PGH2 that is converted to PGE_2_ and PGI_2_ by the action of the enzymes prostaglandin E synthase (PGES) and prostaglandin I synthase (PGIS), respectively. The synthesis of anti-inflammatory prostaglandin E3 (PGE_3_) and prostacyclin I3 (PGI_3_) proceeds through a similar mechanism involving the release of EPA from the cellular membrane, the production of PGH3 (from cyclooxygenase enzyme) that is converted to PGE_3_ and PGI_3_ by the action of PGES and PGIS, respectively. Upon action, PGI_2_ and PGI_3_ are rapidly inactivated to the metabolites 6-keto-PGF_1α_ and Δ17-6-keto-PGF_1α_, respectively, through non-enzymatic hydrolysis processes [3]. The stable products 6-keto-PGF_1α_ and Δ17-6-keto-PGF_1α_ are commonly measured as estimators of the production of prostacyclins (PGI_2_ and PGI_3_, respectively) in biological systems [4].

The physiological properties and pathological effects of prostanoids have been recognized in different biological systems and processes [5], [6], [7], [8]. For example, in fish models prostanoids have been associated with the cardiovascular system [9], the renal system [10], ovulation and spawning processes [11, 12], regulation of branchial ion fluxes [6], osmoregulation process [13], and inflammation [5, 14].

The production of prostanoids by head kidney and liver, tissues and cells, from fish and their involvement in immune modulation and metabolism has been reported elsewhere [15], [16], [17], [18], [19], [20], [21] Head kidney leukocytes and liver cells from salmon have Toll like receptors that are able to respond to foreign antigens and stress by the modulation of eicosanoid production [19, 20]. It has been demonstrated that liver cells are less responsive than head kidney cells and need signals from the highly responsive head kidney cells to properly reveal their inflammatory responses [19, 22]. Head kidney leukocytes from fish play an important role in the proliferation of T-cells through the modulation of prostanoids [23]. Liver is regarded as the site of action for prostaglandins for its ability to bind and degrade them [24], [25], [26]. An unique feature of the fish liver is the constant presence of specific prostanoid-like recepetors with interesting implications for organ function [26]. For instance, studies in fish liver have indicated the involvement of prostaglandins in the cAMP-mediated actions on glycogen and glucose metabolism, and probably other pathways regulated by cAMP [26, 27].

The underlying mechanism behind the production of PGE_2_ or PGE_3_ in fish cell systems exposed to fatty acids has been explained in terms of released ARA or EPA from cell membrane phospholipids and their rapid conversion into their corresponding active metabolites by cyclooxygenases, respectively [15, 28]. Both metabolites, PGE_2_ and PGE_3_, are stable and suitable end-points for studies aiming at monitoring their production in mammalian and fish systems.

Prostacyclins (PGI_2_ and PGI_3_) mediate pro-inflammatory stimuli in non-allergic acute inflammation, while acting as anti-inflammatory mediators [6] and increase vascular resistance of the teleostean gill [29]. In addition, PGI_2_ has been regarded as the major prostanoid synthesized by blood cells from catfish [30]. Studies on mammals have indicated that both metabolites, PGI_2_ and PGI_3_, exhibit equivalent platelet and vascular activity [31], and that PGI_2_ may be involved in ischemic renal disease and chronic renal failure [3, 32].

The synthesis of eicosanoids by different human organs exposed to different stimuli has enabled to understand their physiological function and therapeutic role in inflammation and immunology [7, 8, 33]. Nevertheless, there remains much that it is not known about production of prostanoids in fish organs. The potential of ARA- and EPA-derived prostanoids as biomarkers in fish systems exposed to fatty acids has yet to be realized and can contribute to understand their underlying signaling mechanisms and to improve our knowledge on fish physiology and health. The present research aims at studying the differential synthesis of prostaglandins (PGE_2_ and PGE_3_) and prostacyclins (PGI_2_ and PGI_3_ as their stable metabolites 6-keto-PGF_1α_, and Δ17-6-keto-PGF_1α_, respectively) by salmon cells, isolated from head kidney and liver and exposed to ARA and/or EPA. To the best of our knowledge, this is the first study reporting a predominant production of prostaglandins and prostacyclins by liver and head kidney cells extracted from salmon, respectively.

## 2 Materials and Methods

### 2.1 Reagents

Prostaglandins PGE_2_ (99%) and PGE_3_ (98%); prostacyclins 6-keto-PGF_1α_ (98%) and Δ17-6-keto-PGF_1α_ (98%); deuterated internal standards PGE_2_-d_4_ (99%) and 6-keto-PGF_1α_-d_4_ (99%) were purchased from Cayman Chemical (Ann Arbor, MI, USA). Acetonitrile (99.8%), methanol (99.8%) and formic acid (98%) were purchased from Sigma-Aldrich (St. Louis, MO, USA). A Millipore Milli-Q system was used to produce ultra-pure water 18 MΩ (Millipore, Milford, MA, USA). Cis-5,8,11,14-eicosatetraenoic acid (ARA, 85%) and cis-5,8,11,14,17-eicosapentaenoic acid (EPA, 99%), were purchased from Sigma–Aldrich (Oslo, Norway). Leibovitz`s L-15 medium and laminin (cat#L2020) were from Sigma-Aldrich (St. Louis, MO, USA). Fetal bovine serum (FBS, cat# 14-801F) was from BioWhittaker (Petit Rechain, Belgium). The glutaMaxTM 100 × (cat# 35056) and the collagenase type IV (cat#17104019) were from Gibco-BRL (Cergy-Pontoise, France). The penicillin-streptomycin mixture (cat#17-602E) and the trypan blue solution (cat#17-942E) were from Lonza (Falun, Sweden).

### 2.2 Isolation of the cells from salmon

The cells were isolated from three Atlantic salmon (*Salmo salar*) with a mean body weight of 650 g. The fish were housed by Bergen Aquarium, Norway and fed a commercial diet. The experimental protocol was approved by the Norwegian Board of Experiments with Living Animals. Three biological replicates were used and the cells from every individual fish were submitted to the different treatment as indicated in Table 1.

**Table 1:** A general overview of the experimental protocol indicating the number of biological replicates used to evaluate the production of prostanoids by liver and head kidney cells in the control (without fatty acids), arachidonic acid (ARA), eicosapentaenoic acid (EPA) and their combined fatty acids (ARA+EPA). The tick symbol (✓) represents an individual preparation.

Fish number	Isolated cells	Control	ARA	EPA	ARA+EPA
Salmon#1	Liver	✓	✓	✓	✓
	Head kidney	✓	✓	✓	✓
Salmon#2	Liver	✓	✓	✓	✓
	Head kidney	✓	✓	✓	✓
Salmon#3	Liver	✓	✓	✓	✓
	Head kidney	✓	✓	✓	✓

#### 2.2.1 Liver cells

The fish was anestheticized by metacaine (MS222, 0.5 g/10 L), opened with a sterile scalpel and the exposed liver was slightly lifted to get access to vena porta. The hepatic portal vein was perfused via cannulation (PE50 cannula, BD Venflon Pro, Oslo, Norway) with a perfusion buffer containing EDTA at a flow of 4 mL/min until free of blood. A complete L-15 medium was prepared by mixing Leibovitz`s L-15 medium with 1% glutamax, 1% antibiotic and 10% FBS (cL-15). A solution containing 1 M CaCl_2,_ and a perfusion buffer-I containing 1.4 M NaCl, 0.067 M KCl and 0.09 M Hepes sodium salt at pH 7.4 were prepared and used as stock solutions. A perfusion buffer-II was prepared by adding 1.11 g EDTA disodium salt to 20 mL of the perfusion buffer-I and diluted to 200 mL using ultra-pure water; pH was finally adjusted to 7.4. A perfusion buffer-III was prepared by diluting 10 mL of perfusion buffer-II to 100 mL and adjusting pH=7.4. Afterwards, 100 μL 1M CaCl_2_ and 100 mg collagenase were added. The free of blood liver was digested with collagenase (0.1% collagenase type IV was dissolved in the 0.9 M Hepes buffer as used for perfusion) at room temperature for 5 min and dissolved in the above described perfusion buffer-III. The isolated cells were harvested in 10 mL 10% phosphate-buffered saline buffer (PBS buffer: 0.002M KH_2_PO_4_, 0.02M Na_2_HPO_4_, 0.03M KCl and 0.14M NaCl, pH 7.4) at 5°C, filtrated through a 100 μm mesh cell strainer, washed twice in the PBS buffer at 5°C and resuspended in cL-15 medium before the viability of the isolated cells was assessed. All centrifugations were done by 50 × g for 5 min. The cells were counted using a Bürker chamber and 0.4% trypan blue solution and the viability of the liver cells was above 90% (range: 90.8-94.4%). Sterile equipment and buffers were used to isolate the cells.

#### 2.2.2 Head kidney cells

For each fish, the head kidneys were directly sampled and added PBS at 5°C and then cut with a scissor and squeezed through a 40 µM Falcon cell strainer. The cells were transferred to tubes and centrifuged in a Hettich Zentrifugen, 320 R, at 400 × g for 5 min at 4°C. Cell pellets were resuspended in PBS and layered carefully on top of equal amounts of diluted Percoll in densities 1.08 g/mL and 1.06 g/mL. The tubes were centrifuged at 800 × g for 30 min at 4°C. The cell layer in the interface containing the head kidney leukocytes was collected and the cells were pelleted by centrifugation, 400 × g for 5 min at 4°C. An additional washing step in PBS was performed. The cells were counted using a Bürker chamber and 0.4% trypan blue solution and the viability was above 85%.

### 2.3 Cell cultures

Cell culture plates (Costar, Cambridge, MA) were conditioned by adding 1% laminin (500 μL laminin in 50 mL PBS) 1920 μL/well and kept overnight. A complete L-15 (cL-15) medium was supplemented with 10% foetal bovine serum, 2% pen/strep, 2% glutamaxTM100 × and used to prepare three cL-15 medium solutions containing ARA, EPA or ARA+EPA by attaching the fatty acids to FBS and diluting with cL-15 medium to a concentration level of 50 μM. A control solution was made by adding FBS and ethanol (the solvent used to dissolve the fatty acids) and diluting with cL-15 medium. The initial laminin solution was removed from the plates and ~1.67 × 10^6^ liver cells or ~1 × 10^7^ salmon head kidney cells were cultured into each well containing 2 mL of one of the four cL-15 fatty acid preparations (control, ARA, EPA, or ARA+EPA). The cell culture plates were incubated in a normal atmosphere incubator (Sanyo Electric Company Ltd. Osaka, Japan) at 9°C for 24 h under dark conditions. The four suspensions of cells (one control and three fatty acids) were prepared in triplicate. The medium from the liver cells was collected carefully without disturbing the cells attached to the bottom of the plate and stored at -80°C until solid phase extraction (SPE) followed by liquid chromatography tandem mass spectrometry (LC-MS/MS) quantitative analysis. While the head kidney cells were centrifuged at 50 × g for 5 min at 4°C, the medium collected and stored at -80°C until SPE followed by LC-MS/MS analysis.

### 2.4 Extraction procedure

A slightly modified version of an extraction protocol for quantification published elsewhere was used [34]. Briefly, an aliquot of sample (1 mL) was combined with 175 µl of ethanol containing equal concentrations (45 ng/mL) of PGE_2_-d_4_ and 6-keto-PGF1α-d_4_ followed by 20 µl of acetic acid, vortex-mixed and applied on a SPE column (Agilent, ASPEC Bond Elute C18, 500 mg, 3 mL, USA) previously preconditioned with 2 mL of methanol and 2 mL of water. The cartridge was washed with 4 mL of distilled water and 4 mL of hexane. The analytes were eluted with 1 mL of hexane/ethyl acetate (1:2 v/v), collected into glass tubes and the solvent evaporated under a stream of nitrogen. The dried sample was dissolved in 50 μL of methanol, vortex-mixed 30 s, centrifuged at 1620 × g for 3 min and transferred to an auto sampler vial for LC-MS/MS analysis.

### 2.5 Liquid Chromatography Mass Spectrometry

A LC-MS/MS system (Agilent 6495 QQQ triple quadrupole, Agilent Technologies, Waldbronn, Germany) with an electrospray ionization (ESI) interface and iFunnel ionization was used to quantify the eicosanoids. The ultra-HPLC (UHPLC) system was equipped with a Zorbax RRHD Eclipse Plus C18, 95Å, 2.1 × 50 mm, 1.8 µm chromatographic column. The mobile phase delivered at 0.4 mL/min in gradient mode consisted of ultra-pure water with 0.1 % formic acid (solution A) and an equal volume mixture of acetonitrile and methanol with 0.1 % formic acid (solution B). The solvent gradient was as follows: solution A was reduced from 60 to 5 % from 0.00 to 4.00 min, kept at 5 % between 4.00 and 5.50 min, increased to 60 % between 5.50 and 5.51 min and kept at 60 % between 5.51 and 10.00-min. Mass spectrometric detection was performed by multiple reactions monitoring (MRM) in negative mode. The monitored transitions in percentage of ion counts (%) were: m/z 351→ 333, 315, 271 for PGE_2_; m/z 349 → 331, 313, 269 for PGE_3_; m/z 355→337, 319, 275 for PGE_2_-d_4_; m/z 369→351, 333, 315 for 6-keto-PGF_1α_; m/z 367→349, 331, 313 for Δ17-6-keto-PGF_1α_; m/z 373→355, 337, 319 for 6-keto-PGF_1α_ -d_4_. Although PGD_2_ (retention time 3.6 min) was not determined, the method can discriminate it from PGE_2_ (retention time 3.0 min). The ESI parameters were gas temperature (120°C), gas flow rate (19 L/min), nebulizer pressure (20 psi), sheath gas temperature (300°C), sheath gas flow (10 L/min), capillary voltage (3500V) and nozzle voltage (2000V). The integration of the chromatograms was performed using the MassHunter Qualitative Navigator software (version 8.0). The levels of eicosanoids were estimated by means of the internal standards (PGE_2_-d_4_ and 6-keto-PGF1α -d4) and expressed in ng/mL units.

### 2.6 Data analysis

Analysis of variance (ANOVA) was used to detect significant differences and a Dunnett's test to compare the production of PGE_2_, PGE_3_, 6-keto-PGF_1α_ or Δ17-6-keto-PGF_1α_ at the different experimental conditions (ARA, EPA or ARA+EPA) against their production in the control.

## 3 Results

**Fig. 1:**
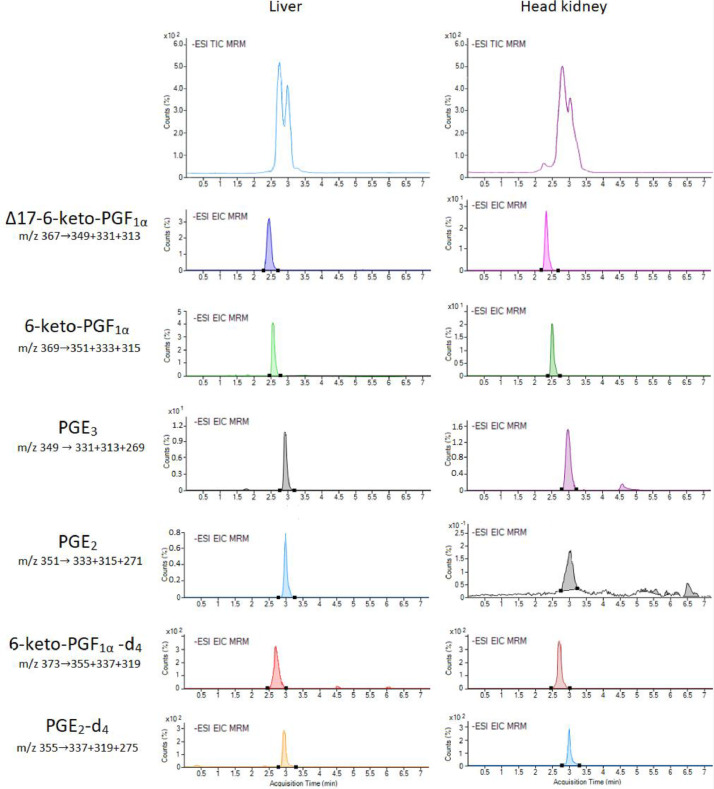
Representative total ion (TIC) and extracted ion (EIC) chromatograms of the targeted prostanoids in the control samples of liver and head kidney cells obtained by using UHPLC-MRM. . The EIC are generated by the summation of the three most intense ions.

The UHPLC system showed an optimal chromatographic performance for all the investigated prostanoids in the controls and fatty acid preparations. Exemplary UHPLC-MRM chromatograms of the targeted prostaglandins and prostacyclins in samples of liver and head kidney cells are provided in Fig. 1.

The production of PGE_2_, PGE_3_, 6-keto-PGF_1α_ (stable metabolite of PGI_2_) and Δ17-6-keto-PGF_1α_ (stable metabolite of PGI_3_) by liver and head kidney cells was evaluated after exposure to 0 or 50 μM of ARA, EPA or ARA+EPA by means of LC-MS/MS analysis and the internal standard technique. The production is reported in Table 2 as average and standard deviation for three biological replicates in concentration units (ng/mL), and their corresponding absolute amounts (picograms). The original data used to compute the different averages in Table 2 are provided in supplementary material (Excel file).

**Table 2:** Levels of synthetized eicosanoids by liver cells and head kidney cells extracted from Atlantic salmon and challenged with different combinations of arachidonic acid (ARA) and eicosapentaenoic acid (EPA). The control consisted of cultured cells without ARA or EPA. All the experiments were performed in triplicate and the results expressed as averages ± standard deviations of three biological replicates (n=3).

		Liver				Head kidney			
		Prostaglandins		Prostacyclins		Prostaglandins		Prostacyclins	
Level		PGE_2_	PGE_3_	PGI_2_	PGI_3_	PGE_2_	PGE_3_	PGI_2_	PGI_3_
ng/mL	Control	0.10±0.00	2.00±0.54	0.23±0.10	0.48±0.20	0.01±0.00	0.15±0.02	2.58±0.97	3.68±1.56
	ARA	2.18±1.02	3.79±1.76	1.16±0.24	3.68±0.67	0.69±0.25	0.51±0.25	14.28±2.54	19.78±2.72
	EPA	0.40±0.01	3.48±1.27	0.48±0.07	1.48±0.39	0.03±0.00	0.18±0.06	0.84±0.30	6.47±1.13
	ARA+EPA	2.33±1.12	6.69±0.08	1.36±0.35	4.28±0.82	0.46±0.06	0.20±0.03	9.48±2.16	14.61±1.37
picograms*	Control	5.1±0.1	99.5±26.9	11.5±5.2	24.1±10,1	0.6±0.1	7.7±1.1	128.8±48.3	184.1±77.8
	ARA	108.9±51.1	189.6±88.1	58.0±11.9	183.8±33.6	34.6±12.7	25.5±12.4	714.0±126.8	988.8±136.0
	EPA	20.0±0.3	174.1±63.6	24.1±3.3	73.9±19.5	1.6±0.1	8.8±3.2	42.2±15.1	323.5±56.5
	ARA+EPA	116.7±55.8	334.5±4.2	68.2±17.3	214.1±41.2	23.0±2.9	10.2±1.6	474.1±108.2	730.5±68.3

* Estimated by using the final dilution volume (50 μL) of the sample.

**Fig. 2:**
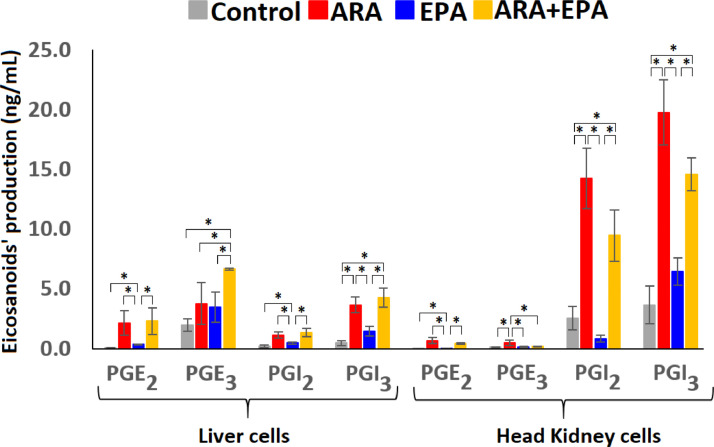
Production of prostaglandins (PGE_2_ and PGE_3_) and prostacyclins (PGI_2_ and PGI_3_) after exposing liver and head kidney cells extracted from Atlantic salmon to different combinations of arachidonic acid (ARA) and eicosapentaenoic acid (EPA). Control cells without ARA or EPA were used for comparison. The bars represent the production expressed as average and standard deviation (µ±σ) for three biological replicates (n=3).

Head kidney cells synthetized considerable higher levels of prostacyclins (PGI_2_+PGI_3_) than prostaglandins (PGE_2_+PGE_3_) and the liver cells exhibited the opposite behaviour (Fig. 2). For instance, the Σprostacyclins/Σprostaglandins ratios for control, ARA, EPA and ARA+EPA were 37.9, 28.4, 35.1 and 36.2 for head kidney cells and 0.3, 0.8, 0.5 and 0.6 for liver cells.

The total production of anti-inflammatory EPA-metabolites (PGE_3_+PGI_3_) were constantly higher than their inflammatory ARA-metabolites counterparts (PGE_2_+PGI_2_) in both, liver and head kidney cells, and in all tested preparations (control, ARA, EPA, ARA+EPA). The ΣEPA-metabolites/ΣARA-metabolites ratios for control, ARA, EPA and ARA+EPA were 7.5, 2.2, 5.6 and 3.0 for liver cells and 1.5, 1.4. 7.6 and 1.5 for head kidney cells.

### 3.1 PGE_2_ production

The systems ARA+EPA and ARA exhibited the highest and similar production of PGE_2_ (2.3±1.1 and 2.2±1.0 ng/mL, respectively) by liver cells. The equivalent PGE_2_ production by head kidney cells were 5.1 and 3.2 times lower than liver cells, respectively. There was no statistically significant difference between these lower values (p>0.05). The comparison between the production of PGE_2_ in the control (0.1±0.0 ng/mL) and the EPA system (0.40±0.0 ng/mL) for liver cells (102.4±2.9 pg/mL vs 401.0±6.8 pg/mL, respectively) and for head kidney cells (11.6±1.1 pg/mL vs 32.4±2.0 pg/mL, respectively) indicated a significant increase in production in both cultures after exposure to EPA. The production of PGE_2_ by liver cells was always higher than head kidney cells in all the assessed systems.

### 3.2 PGE_3_ production

The highest production of PGE_3_ by liver cells was observed in the system ARA+EPA (6.7±0.1 ng/mL) while the systems control, ARA and EPA exhibited similar production of PGE_3_ (2.0±0.5, 3.8±1.8 and 3.5±1.3 ng/mL, respectively). The production of PGE_3_ by head kidney cells in the control or exposed to EPA or ARA+EPA were similar, while in the ARA system was 3-fold higher than the control (0.2±0.0 ng/mL). In general, the production of PGE_3_ by head kidney cells was considerably much lower than liver cells.

### 3.3 PGI_2_ production

The PGI_2_ production by liver cells exposed to ARA (1.2±0.2 ng/mL), EPA (0.5±0.1 ng/mL) or ARA+EPA (1.4±0.3 ng/mL) were significantly different from the control (0.2±0.1 ng/mL). There was not significant difference between the production of PGI_2_ by liver cells in the ARA and ARA+EPA systems (p>0.05). There was not significant difference between the production of PGI_2_ by head kidney cells in the systems ARA and ARA+EPA (14.3±2.5 and 9.5±2.2 ng/mL, respectively). The production of PGI_2_ in these fatty acid systems (ARA and ARA+EPA) was remarkable high compared to the controls for liver (0.2±0.1 ng/mL) and head kidney cells (2.6±1.0 ng/mL). In contrast, exposure to EPA decreased the production of PGI_2_ compared to the head kidney control but did not reach statistical significance.

### 3.4 PGI_3_ production

The highest production of PGI_3_ by liver cells was observed in the system ARA+EPA (4.3±0.8 ng/mL) and ARA (3.7±0.7 ng/mL) with no statistically significant difference between them. The system EPA (1.5±0.4 ng/mL) promoted a significant increase in production of PGI_3_ compared to the liver control (0.5±0.2 ng/mL) but in a lesser extent than ARA and ARA+EPA. The anti-inflammatory PGI_3_ was the most abundant metabolite in all the head kidney culture preparations. The synthetized levels of PGI_3_ by head kidney cells in increasing order of production were 3.7±1.6, 6.5±1.1, 14.6±1.4 and 19.8±2.7 ng/mL for the systems control, EPA, ARA+EPA and ARA.

## 4 Discussion

The control cell preparations indicated that the major products of cyclooxygenase activity are prostaglandins (PGE_3_>PGE_2_) in liver and prostacyclins in head kidney cells (PGI_3_>PGI_2_). The consistent major proportion of EPA (PGE_3_+PGI_3_) than ARA (PGE_2_+PGI_2_) metabolites in both, liver and head kidney cells is mainly due to the inherent highest levels of EPA substrate in these cells compared to ARA substrate. Previous works have consistently confirmed higher levels of the omega-3 than the omega-6 fatty acids in liver (4.9±0.5% EPA and 1.2±0.2% ARA) [35] and head kidney cells (5.5±0.2 EPA and 0.8±0.0% ARA) cells [36] isolated from salmon.

A previous study on the production of PGE_2_ by head kidney cells observed a significant decrease when the cells were exposed to ARA and a significant increase when the cells were exposed to ARA+EPA [28]. The former but not the latter exposure experiment contrasts with the findings of the present study, where both systems (ARA and ARA+EPA) exhibited significant increase in PGE_2_ compared to the controls of both cultured cells (liver and head kidney). The contrasting results for ARA support the conclusions of some researchers that stated that ARA supplementation could not always be ascribed to an increase in prostaglandin synthesis [37] which in turn is an indication that the mechanisms behind the eicosanoid cascade are less straightforward than previously thought.

The increased concentrations of PGE_3_ and PGI_3_ in liver cells (8-fold in ARA and 9-fold in ARA+EPA) and in head kidney cells (5-fold in ARA and 4-fold in ARA+EPA) compared to the controls suggest the incorporation of ARA into the cell membrane at the expense of EPA, a process that has been already reported in mammalian models [38], [39], [40] and might explain the statistically significant increase in production of the EPA derived prostacyclin.

The summation of the total amounts of omega-3 metabolites (Σ[PGE_3_+PGI_3_]) in absolute units were 373.4 pg for liver cells and 1014.2 pg for head kidney cells challenged with ARA (Table 2), and the equivalent amount for the omega-6 metabolites (Σ[PGE_2_+PGI_2_]) were 44.1 pg in liver cells and 43.9 pg in head kidney cells challenged with EPA. These results indicate that unequal amounts of EPA and ARA substrates (EPA>ARA) were released from the cell membranes, at the expense of the incorporation of ARA and EPA, respectively. In addition, these results suggest that the mechanism governing the incorporation of ARA and release of EPA from cell membrane is more effective than the incorporation of EPA and release of ARA. Similar results have been observed in studies where the synthesis of PGE_3_ by head kidney cells exposed to ARA and ARA+EPA was always higher than the synthesis of PGE_2_ [28, 41]. Also, the biosynthesis of PGE_3_ in salmon liver was consistently higher than PGE_2_ and independent of the dietary omega-6/omega-3 ratio that was varied between 0.7 and 4.1 [17]. Dietary studies on salmon and turbot exposed to different omega-6/omega-3 ratios (between 0.6 and 1.2 and 0.1 and 2, respectively) observed a higher incorporation of ARA than EPA in the phosphatidylinositol fraction of head kidney from salmon and liver from turbot and independent of the omega-6/omega-3 ratios [16, 42]. Studies on mammalian models have also concluded that the amount of EPA incorporated into lipids is always less than the ARA that is replaced [39].

Estimated (ARA+EPA)/ARA concentration ratios of 1.07 (PGE_2_) and 1.17 (PGI_2_) for liver cells and of 0.67 (PGE_2_) and 0.66 (PGI_2_) for head kidney cells might indicate similar incorporation rate of ARA into the cell membranes in both formulations, (single or combined) and similar affinity of both synthases (PGES and PGIS) for ARA towards the production of PGE_2_ and PGI_2_. In addition, the computed ratios over 1.0 for liver cells suggests a constant production of PGE_2_ and PGI_2_ regardless of the presence of EPA, while a ratio over 0.6 for head kidney cells might suggest that EPA supplementation may lead to a shunt of the ARA metabolism through the cyclooxygenase pathway and the consequent decrease of PGE_2_ and PGI_2_.

It has been pointed out that exposure to EPA decreases ARA concentration by releasing it from the cell membrane phospholipids [43, 44]. The released ARA is then rapidly converted into active metabolites by cyclooxygenases to produce prostaglandins, prostacyclins, and thromboxanes, and by lipoxygenases to produce leukotrienes [44]. The amounts and types of synthesized eicosanoids are dictated by several factors, such as the availability of ARA, the cell type and the enzymatic activity of both, cyclooxygenase and/or lipoxygenase [45]. Although the previous observations have been derived from mammalian models, they seem a valid potential mechanism to explain the increased production of PGE_2_ by factors of ~4 in liver and ~3 in head kidney cells exposed to EPA compared to the controls.

The observed inhibition of PGI_2_ formation in head kidney cells by EPA has been also observed in mammalian cells, where the enrichment of endothelial cell with EPA decreases PGI_2_ generation, probably due to a decreased liberation of endogenous ARA from lipid stores [46].

Interestingly, the results of the present work indicating that ARA significantly stimulates PGI_3_ production in both cell systems (liver and head kidney) have been also observed in a human study where exposure to ARA strongly stimulated PGI_3_ production by endothelial cells [46] and markedly increases the cyclooxygenation and lipoxygenation of EPA in human platelets [47].

The low production of prostaglandins by head kidney cells in the present study resembles the behaviour of human embryonic kidney cells transfected with PGES, and where a low PGE_2_ synthesis was observed [48, 49]. High prostacyclin to prostaglandin ratios (PGI_2_/PGE_2_ and PGI_3_/PGE_3_) have been also recorded in most experimental models following cyclooxygenase (COX-2) induction [50], [51], [52], [53], [54], [55]. It is well documented that COX-2 is responsible for most of the PGI_2_ (and to a smaller extent of PGE_2_) production [56]. The mechanism of action of increased prostacyclins remains to be determined.

Altogether the present research supports the following production pattern (PGE_3_+PGI_3_)>(PGE_2_+PGI_2_) for cell systems with high endogenous EPA/ARA ratio. It is likely that a low endogenous EPA/ARA ratio will change the direction of the inequality into (PGE_3_+PGI_3_)<(PGE_2_+PGI_2_). This observation is confirmed by analysing the results of a human cell system with low endogenous EPA/ARA ratio that was exposed to ARA, EPA and ARA+EPA [46], and where the estimated production pattern of (PGE_2_+PGI_2_)>(PGE_3_+PGI_3_) was opposite to that observed in the present study. In addition, a recent study on the production of eicosanoids and the content of EPA and ARA in liver from Atlantic salmon, observed that high EPA/ARA ratios in liver phospholipids were correlated with significant increase in PGE_3_ concentration. For instance, EPA/ARA ratios of 0.6 and 2.3 correspond to 0.3±0.1 and 1.9±0.6 ng/mL of PGE_3_, respectively [17].

The present study indicates that the predominant metabolites produced by liver and head kidney cells from Atlantic salmon are prostaglandins (PGE_3_>PGE_2_) and prostacyclins (PGI_3_>PGI_2_), respectively. The supplementation of ARA might modulate the production of PGI_3_ and PGE_3_ by releasing EPA from the liver and head kidney cell membranes rather than compete with it for the same cyclooxygenase or lipoxygenase enzymes, which pointed out the complexities associated with unraveling the factors and mechanisms responsible for eicosanoid production.

The predominant production of prostacyclins by head kidney cells from salmon has not been reported previously. It remains to be seen whether the immune responses of head kidney cells isolated from salmon are regulated via prostacyclins.

The most significant finding in the present research is the differential production of prostaglandins (PGE_2_, PGE_3_) and prostacyclins (PGI_2_, PGI_3_) by liver and head kidney cells from salmon in response to ARA and EPA exposure. Therefore, it is likely that the effect of these metabolites will rely on their levels in a specific cell, yielding a specific and distinct physiological response which will enable to understand their role in inflammation and immunology.

## Declaration of Competing Interest

The authors declare that they have no known competing financial interests or personal relationships that could have appeared to influence the work reported in this paper.

## Appendix Supplementary materials

Image, application 1
